# Protocol for endolysosomal proteomics in goat atrial tissue using a refined density-gradient approach

**DOI:** 10.1016/j.xpro.2025.104248

**Published:** 2025-12-08

**Authors:** Thamali Ayagama, Georgina Berridge, Roman Fischer, Rebecca A. Capel, Sander Verheule, Ulrich Schotten, Rebecca A.B. Burton

**Affiliations:** 1 Department of Pharmacology, University of Oxford, Oxford, UK; 2 Department of Physiology, Anatomy and Genetics, University of Oxford, Oxford, UK; 3 Target Discovery Institute, University of Oxford, Oxford, UK; 4 Departments of Physiology and Cardiology, Cardiovascular Research Institute Maastricht, Maastricht University, Maastricht, the Netherlands; 5 University of Liverpool, Department of Pharmacology and Therapeutics, Institute of Systems, Molecular and Integrative Biology, Liverpool, UK; 6 Liverpool Centre for Cardiovascular Science, University of Liverpool and Liverpool Heart & Chest Hospital, Liverpool, UK

**Keywords:** Biotechnology and bioengineering, Cell Biology, Cell separation/fractionation, Mass Spectrometry

## Abstract

Here, we present a protocol to analyze endolysosomal (EL) proteins in goat atrial fibrillation (AF) tissue using a modified density-gradient, proteomic-based fractionation. This method separates organelles from tissue lysate, isolating sarcoplasmic reticulum, mitochondria (1.3 g/mL), and EL (1.04 g/mL) with minimal contamination. The protocol includes tissue isolation, homogenization, density-gradient processing, peptide analysis, data analysis, and protein network identification using discontinuous Percoll and sucrose gradients; it supports molecular and proteomic studies.

For complete details on the use and execution of this protocol, please refer to Ayagama et al.^1^ and Ayagama et al.^2^

## Highlights

• Refined Percoll-sucrose gradient for organelle separation from goat atrial lysate
• Guidance on preserving organelle integrity, enabling EL proteomics for protein discovery
• Procedure for tissue preparation, fractionation, and peptide and EL proteomic analysis

## Abstract (editor highlights)

Publisher’s note: Undertaking any experimental protocol requires adherence to local institutional guidelines for laboratory safety and ethics.

## Graphical abstract

**Figure:**
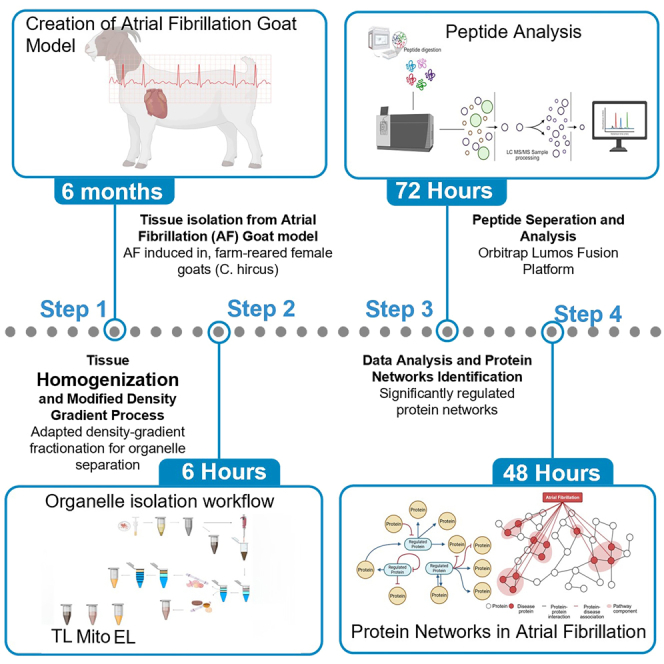

## Teaser

Here, we present a protocol to analyze endolysosomal (EL) proteins in goat atrial fibrillation tissue using a modified density-gradient, proteomic-based fractionation. This method separates organelles from tissue lysate, isolating sarcoplasmic reticulum, mitochondria (1.3 g/mL), and EL (1.04 g/mL) with minimal contamination. The protocol includes tissue isolation, homogenization, density-gradient processing, peptide analysis, data analysis, and protein network identification using discontinuous Percoll and sucrose gradients; it supports molecular and proteomic studies.

## Before you begin

### Innovation

Density gradient methods for isolating endolysosomes from other organelles such as mitochondria and the ER/SR are inherently challenging due to overlapping buoyant densities that hinder clean separation. Earlier protocols commonly relied on sucrose gradients, which can induce osmotic stress and organelle swelling when samples remain at differential densities for extended periods. As a result, isolated organelles were frequently functionally compromised, limiting their utility in downstream metabolic and signaling assays. Moreover, these methods predated the integration of proteomic analysis, restricting organelle identification to indirect enzyme activity assays that lacked molecular specificity.

In our modified approach, we employ Percoll-based gradients to minimize osmotic damage and preserve organelle integrity and function. By coupling this refined isolation strategy with proteomic profiling, we achieve comprehensive identification of multiple endolysosomal markers and delineate their functional relevance in AF, offering new insight into organelle-specific dysfunction and its contribution to disease mechanisms.

### Institutional permissions

The AF Goat Model Study was carried out in accordance with the principles of the Basel Declaration and regulations of European directive 2010/63/EU, and the local ethical board for animal experimentation of the Maastricht University approved the protocol. To duplicate this method in an animal model, the researchers must acquire permissions from relevant institutions.

### Preparation

#### Tissue extraction and homogenization

1. Induce and maintain AF in N=4 farm-reared female goats (*Capra hircus*) for 6 months.

***Note:*** To obtain comparative data, include N=4 sham (control) farm-reared female goats (*Capra hircus*). The animals are 24 to 34 months and weigh 72±8 kg. Please refer to van Hunnik et al.^3^ for the complete protocol.

***Note:*** In brief, surgically implant electrodes on the pericardium above the left atrium of the goats under anesthesia (sufentanil and propofol). After a 2-week recovery period, induce AF using a subcutaneous neurostimulator and maintain it for 3–4 weeks. For terminal open-chest experiments, anesthetize the animals with sufentanil, propofol, and rocuronium.

2. After euthanizing the animals, perfuse the hearts with saline to clear blood and collect left atrial biopsies from a consistent atrial region.

***Note:*** Atrial tissue biopsies from both AF and sham goats need thorough washing with phosphate-buffered saline (PBS). A minimum of 100 mg of tissue per sample is required for proteomic analysis.

***Note:*** Use freezer-safe vials, place each tissue biopsy in a vial and label them. Collect eight biopsies (one biopsy) from each animal for this experiment. Snap-freeze the vials containing the tissue biopsies in liquid nitrogen as soon as the tissue collection takes place, transfer the vials carefully for further storage into a −80°C freezer.

**CRITICAL:** Tissue should be snap frozen within seconds to avoid decomposition of the sample.

## Key resources table

**Table:** 

REAGENT or RESOURCE	SOURCE	IDENTIFIER
**Biological samples**		

Left atrial tissue from *C. hircus* atrial fibrillation models (farm-reared female goats; age: 24 and 34 months; and weight: 72±8 kg)	Maastricht University, Netherlands	*C. hircus*

**Chemicals, peptides, and recombinant proteins**		

Lysosome Isolation Buffer	BioVision	K235-50-1
Lysosome Enrichment Buffer	BioVision	K235-50-2
Protease Inhibitor Cocktail (PIC/PHI)	BioVision	K235-50-4
Phosphate-buffered saline (PBS)	Gibco PBS (Thermo Fisher)	10010023
Methanol	Thermo Fisher Scientific	10636545/CAS67-56-1
Chloroform	Thermo Fisher Scientific	AC149830010/CAS67-66-3
Trypsin MS Grade	Thermo Fisher Scientific	90058/CAS9002-07-7
Sucrose	Fisher Scientific	15503022/CAS57-50-1
Percoll	Santa Cruz Biotechnology	sc-500790/CAS9009-32-7
Dithiothreitol	Merck (Sigma-Aldrich)	D0632/CAS3483-12-3
Urea	Merck (Sigma-Aldrich)	51456/CAS57-13-6
Acetonitrile	Merck (Sigma-Aldrich)	100029/CAS 75-05-8
Dimethyl sulfoxide (DMSO)	Thermo Fisher Scientific	85190/CAS67-68-5
Formic acid	Thermo Fisher Scientific	A117-50/CAS64-18-6
TRIS HCL	Merck (Sigma-Aldrich)	4103-01/CAS1185-53-1
Iodoacetamide	Merck (Sigma-Aldrich)	A39271/CAS144-48-9

**Deposited data**		

Mass spectrometry proteomics data, Proteome Xchange via PRIDE partner repository, dataset identifier PRIDE Database: PXD041056	Ayagama et al.^1^	Database: http://proteomecentral.proteomexchange.org/cgi/GetDataset?ID=PXD041056
A modified density gradient proteomic-based method to analyze endo-lysosomal proteins in cardiac tissue	Ayagama et al.^2^	https://www.cell.com/iscience/fulltext/S2589-0042(21)00917-2

**Experimental models: Organisms/strains**		

Farm-reared female goats (*C. hircus*); age: 24 and 34 months; and weight: 72±8 kg.	Maastricht University, Netherlands	N/A

**Software and algorithms**		

Progenesis QI software platform (version 4.2)	Waters Cooperation	www.nonlinear.com
Perseus software platform (version 1.6.15.0)	Tyanova et al.,2016	http://coxdocs.org/doku.php?id=perseus:start
STRING Network analysis	N/A	STRING Database Version 11/string-db.org

**Other**		

Dionex Ultimate 3000 RSLC system coupled to an Orbitrap Fusion Lumos platform	Thermo Fisher Scientific	N/A
TLX Beckmann Coulter Ultra Centrifuge	Beckman Coulter	N/A
Thermo Scientific Heraeus Pico 17 tabletop centrifuge	Thermo Fisher Scientific	N/A
SpeedVaccum centrifuge	Thermo Fisher Scientific	N/A
EASY-Spray column	Thermo Fisher Scientific	ES803
Sola-HRP-SPE cartridges	Thermo Fisher Scientific	60109-001

## Step-by-step method details

### Tissue homogenization

**Figure 1:**
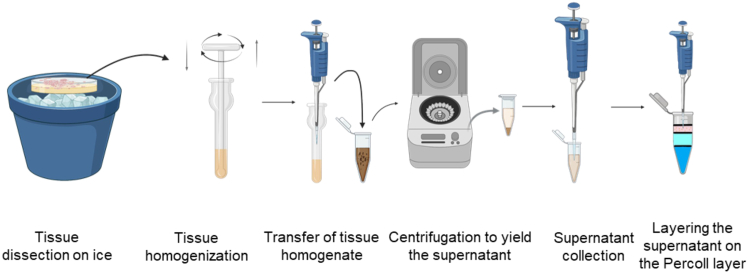
Overview of the preparation workflow Tissue samples were dissected on ice, homogenized, and centrifuged to generate a supernatant. The supernatant was then collected and applied to a Percoll layer for further separation.

**Figure 2:**
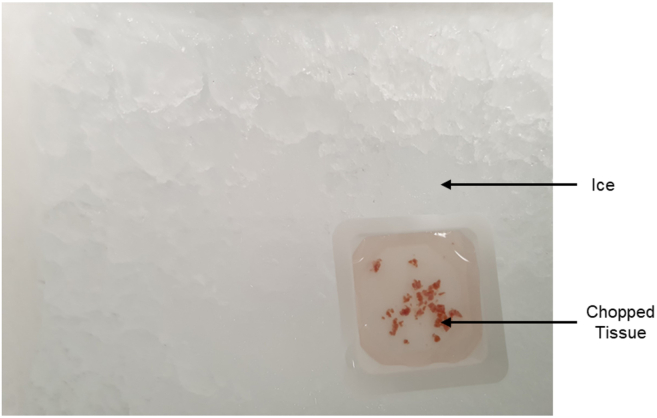
Dissected goat atrial tissue, finely chopped and maintained on ice to preserve integrity during sample preparation

***Note:*** Clean the micro-scissors and the tissue holding dish between each tissue biopsy to avoid cross contamination of the samples. Label eight Eppendorf tubes and transfer the chopped tissue into each tube.

1. On the day of the tissue preparation for density gradient-separated fractionation:
  a. Place a glass-petri dish on ice and pour ice-cold PBS in the petri-dish.
  b. Thoroughly clean each atrial tissue biopsy for **10 s** one by one with ice-cold PBS. Clean the glass petri-dish between handling each tissue biopsy.
  c. Chop the cleaned tissue with the help of sterile scalpels and dissection micro-scissors (Figures 1 and 2).

2. Tissue homogenization:
  a. Begin homogenization with a 7 mL Dounce homogenizer with a loose pestle.
  b. Add 500 μL Lysosome isolation buffer (LIB) containing [protease inhibitor cocktail (PIC) and phosphatase inhibitor (PHI) 1:500 ratio].
    ***Note:*** Use gentle strokes until the tissue disintegrates and creates a homogenate.
  c. Transfer the lysate to a 1 mL Dounce homogenizer with a tight pestle for further homogenization.
    ***Note:*** Continue this procedure for each sample.
    **CRITICAL:** Ensure that throughout the homogenization, the Dounce homogenizer and the tissue are placed on ice (Figure 1).

### Percoll-sucrose gradient setup and organelle collection workflow

3. Transfer the lysate (step 2) to chilled 1.5 mL Beckmann Coulter Ultracentrifugation tubes and mix with Lysosome enrichment buffer (LEB).

***Note:*** Use Biovision LEB containing 1:500 PIC at a 1:1.5 ratio with the lysate. Use more tubes if required.

4. Gently invert the tubes to allow the LEB to mix with the supernatant to create a homogenate.

Incubate the homogenate on ice for **5 minutes** (min).

5. Briefly centrifuge the homogenate at 13,000 g for 2 min at 4°C, using a TLX Beckmann Coulter Ultra Centrifuge. Carefully collect each of the supernatants into eight new Eppendorf tubes without disturbing the tissue pellet.

***Note:*** Label the supernatant as tissue lysate (TL) (Figure 1).

**Figure 3:**
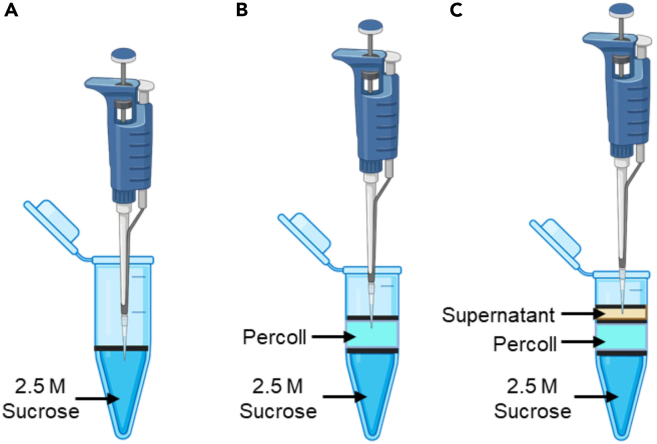
Stepwise layering in a 1.5 mL ultracentrifuge tube (A) 2.5 M sucrose at the base, (B) Percoll carefully layered above the sucrose, and (C) tissue supernatant added on top.

***Note:*** Label sixteen Eppendorf tubes, eight of them to collect the mitochondria and SR rich fraction (label them Mito), and the remaining eight to collect the endolysosome fraction (label them EL).

**Figure 4:**
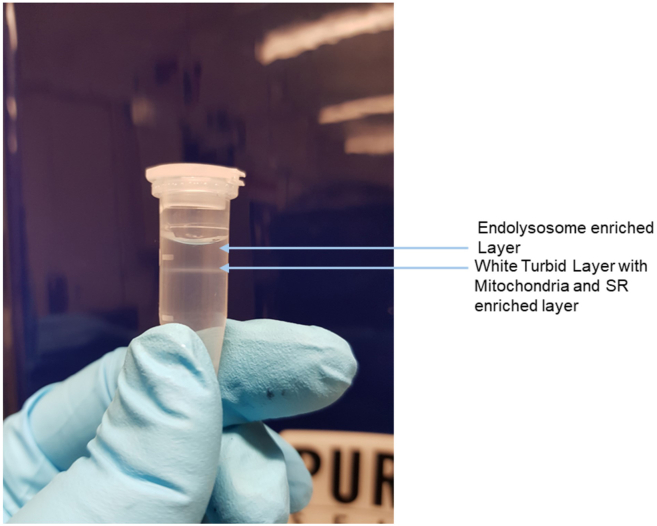
Representative ultracentrifuge tube following separation on a Percoll sucrose gradient Arrows indicate the mitochondrial/sarcoplasmic reticulum (SR) enriched fraction and at the top the endo lysosomal (EL) enriched fraction, that require further purification steps.

6. Pipette 750 μL of 2.5 M sucrose into a 1.5 mL ultracentrifuge tube, followed by 250 μL of Percoll on top of the sucrose layer. Then, carefully pipette 200 μL of TL (Figure 3) and centrifuge at **27,000 g-force, 10°C for 50 min.**

7. Observe the white turbid layer (that contains mitochondria and SR) at the biface of Percoll and 2.5 M sucrose (Figure 4). Using a 200 μL pipette, carefully collect this layer containing mitochondria and SR-enriched fraction into the eight Eppendorf tubes previously set aside labeled Mito.

**CRITICAL:** Collection of this layer should be done carefully to avoid contamination from other layers.

**Figure 5:**
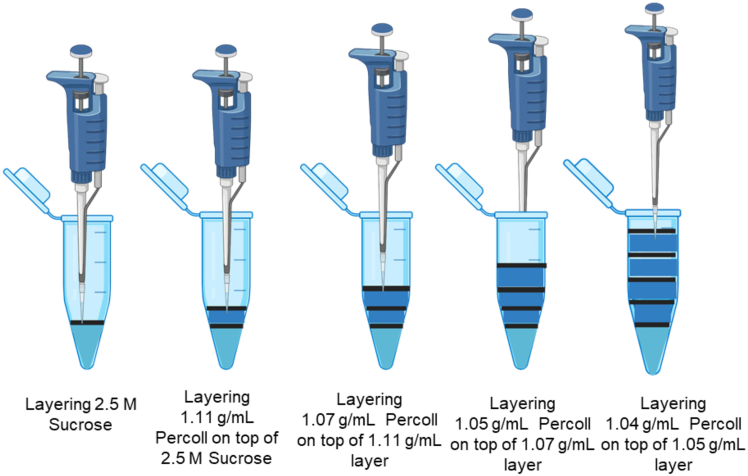
Schematic representation of density gradient preparation for differential centrifugation Ultra-centrifuge tubes were sequentially loaded with 2.5 M Sucrose and Percoll solutions of decreasing density (1.11 g/mL, 1.07 g/mL, 1.05 g/mL, and 1.04 g/mL).
  b. Underlay with 2.5 M Sucrose at the bottom of each of the eight tubes and carefully overlay with a series of Percoll dilutions in ddH_2_O in order: 1.11 g/mL, followed by 1.07 g/mL, followed by 1.05 g/mL, and finally 1.04 g/mL (Figure 5).
13. Gently pipette the fraction right above the faint turbid-white layer of the centrifuged tubes from step 11, and transfer each collected fraction, one by one, onto a newly prepared Percoll gradient tube from step 12.

***Note:*** Follow this step for all 8 tubes.

8. Collect the layer above the turbid white layer that contains endosomes and endolysosomes with minimum contamination of SR (Figure 4).
9. Place the newly prepared tubes on ice and carefully transfer the crude endosomal and EL fractions from step 6, to the labeled Eppendorf tubes.
10. Prepare another set of 8 x 1.5 mL ultracentrifuge tubes.
  a. Layer 500 μL of 2.5 M Sucrose and 500 μL Percoll in each.
  b. Using a 200 μL pipette carefully layer 100 μL of the collected EL fraction on top of these newly prepared 1.5 mL ultracentrifuge tubes.
11. Ultracentrifuge the tubes at **29,000 g-force set at 15°C for 30 min** to remove any remaining SR organelles.
12. Once the tubes are centrifuging, prepare a new set of 8 x 1.5 mL Eppendorf tubes for the differential density gradient centrifugation step.
  a. Load the ultra-centrifuge tubes with the 2.5 M Sucrose and different density gradients consisting of, Percoll dilutions (starting from 1.11 g/mL, 1.07 g/mL, 1.05 g/mL, and 1.04 g/mL) (Figure 5).

14. Ultracentrifuge the eight tubes at **67,000 g-force for 30 min at 4°C.**
15. Collect the topmost fraction containing EL using a 200 μL pipette into eight Eppendorf tubes (Figures 4 and 6).

**Figure 6:**
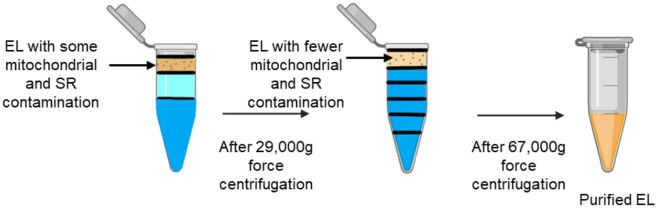
Differential centrifugation workflow for enrichment of endo lysosomes (EL) The initial EL fraction contains some mitochondrial and sarcoplasmic reticulum (SR) contamination. After centrifugation at 29,000 × g, EL fractions show reduced mitochondrial and SR contamination. Following centrifugation at 67,000 × g, a purified EL fraction is obtained.
16. Label and snap freeze the final tubes of TL, Mito and EL in liquid nitrogen and then store them at −80°C.

**CRITICAL:** Steps 1 to 16 of the step-by -step method details, need to be completed in one block and takes approximately **6 h** to complete. These are the most crucial steps to separate and collect the organelle fractions with minimal cross contamination.

**Precautions:** Prepare all the requirements before starting to process the tissue and downstream work flow.

### Identification of EL protein expression using quantitative proteomic analysis

17. Prepare the final samples, TL, Mito and EL from step 16 for Liquid chromatography-tandem mass spectrometry analysis (please refer to^1^ for methodology details). A liquid chromatography system workflow is provided separately (See LC-MS MS System Work flow document).
  a. Add 5 μL of 200 mM dithiothreitol to each sample and incubate for **30 min** at room temperature (RT).
  b. Add 20 μL of 200 mM iodoacetamide to each sample and incubate at RT for **30 min.**
18. Proceed for methanol-chloroform precipitation.
  a. Use 200 μL of sample volume.
  b. If the volume is higher than 200 μL, conduct the process in multiple tubes.
  c. If the sample is less than 200 μL, bring the volume up to 200 μL with double distilled water. To each sample tube add:
  d. 600 μL methanol.
  e. 150 μL chloroform and vortex.
  f. 450 μL MilliQ-double distilled water and vortex for 1 min.
  g. Centrifuge the samples at **13,000 rpm** maximum speed in a Thermo Scientific Heraeus Pico 17 tabletop centrifuge at **RT for 1 min** until the sample appears transparent.
    ***Note:*** Centrifuge longer if the sample is turbid in color.
  h. Observe the precipitate and pipette off the upper aqueous phase without disrupting the precipitate.
  i. Pipette 450 μL methanol to the same sample tube (from step 18h) containing the remaining organic phase (with precipitate) and vortex.
  j. Centrifuge the sample tubes at **13,000 rpm at RT for 2 min** and discard the supernatant.
19. Resuspend the pelleted proteins adding 6 M Urea in 400 mM Tris-HCl, at pH 7.8.
20. Add 250 μL of 400 mM Tris-HCl to the protein sample to dilute the mixture from 6 M Urea to 1 M.
21. Add trypsin to each sample tube at a 1:50 volume ratio and incubate on a rocker at **37°C overnight** to break down the proteins into peptides.
22. After **18 h**, acidify the samples to a final concentration of 1% formic acid to terminate the trypsin digestion.
23. Use Sola-HRP-SPE cartridges (Thermo Fisher Scientific, catalogue no: 60109-001) to desalt the samples.

***Note:*** To perform Sola-HRP-SPE desalting, condition the Cartridges to activate the hydrophobic reversed-phase polymer using 100% acetonitrile. Allow acetonitrile to pass through by gravity or gentle vacuum.

To prepare the sorbent for peptide binding, add 0.1% formic acid. Allow it to pass through completely.

Load each sample slowly onto the cartridge. Allow full binding by gravity or gentle vacuum.

Wash using 0.1 % formic acid to remove salts and hydrophilic impurities. Elute bound peptides with acetonitrile. Collect the eluate in a clean LC-MS vial or low protein-binding microcentrifuge tubes.

24. Dry down the samples using a SpeedVaccum centrifuge.

***Note:*** To dry down the samples ensure the samples are in LC-MS vials or in low protein-binding tubes. Volume should be less <1 mL per tube for optimal evaporation. Avoid sealing the tubes tightly, or use vented caps. Load the rotor by placing tubes evenly in it to maintain balance. For drying fewer samples, use counterbalance tubes with an equal volume of water or buffer to balance the rotor. Turn on the vacuum pump and allow pressure to drop. Then run the drying cycle. Monitor the progress through the transparent lid or system display.

25. Further desalt the samples using PepMAP C18, 300 μm, 5 mm, and 5 μm particles (Thermo Fisher Scientific) at a flow rate of **20 μL/min for 1 min**, and separate peptides on an EASY-Spray column (PepMAP C18, 75 mm 3500mm, 2mm particles, ES803, Thermo Fisher Scientific), **over 60 min** using a 2%–35% acetonitrile gradient in 5 % DMSO, 0.1 % formic acid at 250 nL/min.
26. Using the standard parameters perform the peptide separation and peptide analysis on an Ultimate 3000 UHPLC system coupled to an Orbitrap Fusion Lumos platform (both from Thermo Fisher Scientific).^4^ Refer to LC-MS MS System Work flow document.

***Note:*** Steps 17–26 need to be completed in one block and will take a minimum of **72 h**. Steps 17–26 provide details on how to digest the proteins to peptides after the protein isolation, and the analysis of these peptides.

## Expected outcomes

This protocol provides a refined Percoll-sucrose density gradient method for clean separation of organelles from goat atrial tissue lysate, preserving organelle integrity and enabling identification of EL-specific protein networks associated with AF. It includes detailed steps for tissue preparation, gradient fractionation, peptide processing, and data analysis to facilitate reproducible EL proteomics in cardiac tissue. The modified technique ensures minimal cross-contamination and can be adapted for molecular and proteomic studies in other tissue types.

## Quantification and statistical analysis

Full quantification and statistical analysis can be reviewed in Ayagama et al.^1^ In summary, the analysis of the raw Mass Spectrometry data is performed using the Progenesis QI software platform.

(WatersTM Cooperation, www.nonlinear. Com (version 4.2)). In brief, quantitative proteomic analysis was conducted in Perseus software platform22 (v1.6.15.0) using protein intensity values from biological replicates. After filtering out proteins with excessive missing values, 2,104 proteins from TL and EL samples remained. Data were log2-transformed, Z-score normalized, and analyzed without imputation since signal absence reflected noise. Principal component analysis was performed on complete datasets, and differential protein regulation between AF and sham conditions was assessed using volcano plots with two-way Student’s t-tests. Significance was determined by permutation-based FDR (5%, 250 randomizations, S0 = 0.1), ensuring 99% confidence in quantified proteins.

1. Database searches are performed against the UniProt *C. hircus* database (UP000291000).
  a. Then select the automatic processing or automatic retention time alignment, which is integral to the Progenesis software, to the function suitability and runs to align them.
  b. Select the Peak picking between 10 and 75 min.
2. Quantify the statistical analysis of the data retrieved from Progenesis using the Perseus software platform (version 1.6.15.0). Please refer to Ayagama et al.^1^, for step-by-step data quantification guide.
3. Apply the most significantly regulated protein list to the STRING Network analysis (STRING Database Version 11/string-db.org).
4. Apply the analysis parameters for interaction evidence of each protein to be:
  a. published databases, experiments, gene fusions, co-occurrences and co-expressions.
  b. medium interaction score.
5. To study the network of the original EL proteins discovered from the protocol, use only the query proteins list and avoid applying external interactors.

***Note:*** The analysis stage is not time dependent and can be run over **48 h to days or weeks**. The analysis for protein networks steps is vital to understand the organelle protein dysregulations in the AF.

## Limitations

The Protein network analysis for the identified list of proteins/genes obtained from this protocol is based on published literature and goat vs human comparative databases. The molecular pathways generated provide a fundamental understanding of the disease mechanisms. Because protein network tools periodically update their data feeds, newly discovered information is continuously integrated into the molecular pathways, making each dataset unique and providing an updated set of results (Note: The automatic updating of the protein networks is not a limitation but an important factor to take into consideration).

## Troubleshooting

### Problem 1

Variability in tissue biopsy sampling across different regions in biological replicates may influence the results (Preparation).

### Potential solution

Excise and dissect tissue from the same anatomical region across all biological replicates to ensure consistency (Preparation). Create a standard Operating Procedure (SOP) that defines the exact anatomical landmark(s) (e.g., “2 cm lateral to the left atrial appendage”) and sample size/depth. Use a simple template or diagram in the SOP that technicians follow during excision. Take a photo of each excised site with a ruler and save with the sample ID.

### Problem 2

Drastic changes in the quantity of the starting material can affect the outcome of the protocol. (tissue homogenization).

### Potential solution

To ensure reproducibility and consistent outcomes, perform the protocol several times with the recommended amount of tissue. Limit the sample quantified to that specified and tested in this protocol.

### Problem 3

A few early endosomes enter the EL fraction at the fractionation phase (Step 14).

### Potential solution

• Complete exclusion of early endosomes from the EL fraction is unlikely; however, monitoring early endosomal markers at the proteomic stage serves as a useful quality control step.
• Some of the other post-isolation validation methods include Western blotting and enzymatic assays of the proteins of interest to confirm the purity and integrity.

### Problem 4

Low protein yield from the proteomic workflow (Step 23).

### Potential solution

Avoid over drying samples in the SpeedVac centrifugal step. Partial drying followed by immediate reconstitution can preserve labile peptides.

## Resource availability

### Lead contact

Further information and requests for resources and reagents should be directed to and will be fulfilled by the lead contact, Rebecca A.B. Burton (r.a.b.burton@liverpool.ac.uk).

### Technical contact

Technical questions on executing this protocol should be directed to and will be answered by the technical contact, Thamali Ayagama (thamali.ayagama@dpag.ox.ac.uk).

### Materials availability

This study did not generate new unique reagents. Goat material is not available for this study due to the nature of the study and ethical constraints. Data related to this study can be found at Database: http://proteomecentral.proteomexchange.org/cgi/GetDataset?ID=PXD041056.

### Data and code availability

No new code was generated in this study. All datasets and analysis generated and analyzed can be accessed using the Pride accession PRIDE Database: PXD041056.

## Acknowledgments

R.A.B.B. was funded by a Sir Henry Dale Fellowship, ‘Optical Interrogation of Sub-Cellular Cardiac Signalling in Atrial and Sino-Atrial Node Arrhythmias At High-Spatiotemporal Resolution’ (109371/Z/15/Z), and R.A.B.B. acknowledges support from the Returning Carers’ Fund (Oxford University, Medical Sciences Division). T.A. acknowledges the Global Challenges Research Fund, University of Oxford. R.A.B.B. was a Senior Research Fellow at Linacre College. R.A.B.B. acknowledges research funds from the Ellis T Davies Fellowship Endowment, University of Liverpool. The goat model work was supported by the Netherlands Heart Foundation (CVON2014-09, RACE V Reappraisal of Atrial Fibrillation: Interaction between hypercoagulability, Electrical remodeling, and Vascular Destabilisation in the Progression of AF, and grant number 01-002-2022-0118, EmbRACE: Electro-Molecular Basis and the Rapeutic management of Atrial Cardiomyopathy, fibrillation and associated outcomEs) and the European Union (CATCH ME: Characterizing Atrial fibrillation by Translating its Causes into Health Modifiers in the Elderly, grant number 633196; MAESTRIA: Machine Learning Artificial Intelligence Early Detection Stroke Atrial Fibrillation, grant number 965286). This research was funded in whole, or in part, by the Wellcome Trust (109371/Z/15/Z). For the purpose of Open Access, the author has applied a CC BY public copyright license to any Author Accepted Manuscript version arising from this submission. Proteomics was performed in the Discovery Proteomics Facility of the Target Discovery Institute, University of Oxford. The graphical abstract was created with BioRender.com.

## Author contributions

R.A.B.B. conceived and designed the study; S.V. and U.S. performed the goat AF experiments and provided the tissue for this study. T.A. performed experimental protocol design and validation work, statistical analysis of proteomics, and pathway analysis. R.A.C. and T.A. contributed to protocol optimization and troubleshooting during method development. G.B. and R.F. performed LC-MS/MS. All authors contributed to the writing of the manuscript. T.A. created the graphical figure.

## Declaration of interests

The authors declare no competing interests.

## Declaration of generative AI and AI-assisted technologies in the writing process

During the preparation of this work, the authors used Grammarly for editing suggestions (e.g., spelling, punctuation, and grammar). After using this tool/service, the authors reviewed and edited the content as needed and take full responsibility for the content of the publication.

## Supplemental information

Data S1. LC-MS MS system work flow

## References

[1] Ayagama T., Charles P.D., Bose S.J., Boland B., Priestman D.A., Aston D., Berridge G., Fischer R., Cribbs A.P., Song Q. (2024). Compartmentalization proteomics revealed endolysosomal protein network changes in a goat model of atrial fibrillation. iScience.

[2] Ayagama T., Bose S.J., Capel R.A., Priestman D.A., Berridge G., Fischer R., Galione A., Platt F.M., Kramer H., Burton R.A.B. (2021). A modified density gradient proteomic-based method to analyze endolysosomal proteins in cardiac tissue. iScience.

[3] van Hunnik A., Zeemering S., Podziemski P., Simons J., Gatta G., Hannink L., Maesen B., Kuiper M., Verheule S., Schotten U. (2018). Stationary Atrial Fibrillation Properties in the Goat Do Not Entail Stable or Recurrent Conduction Patterns. Front. Physiol..

[4] Liang Z., Damianou A., Vendrell I., Jenkins E., Lassen F.H., Washer S.J., Grigoriou A., Liu G., Yi G., Lou H. (2024). Proximity proteomics reveals UCH-L1 as an essential regulator of NLRP3-mediated IL-1β production in human macrophages and microglia. Cell Rep..

