# Supplementary material for: Protocol for endolysosomal proteomics in goat atrial tissue using a refined density-gradient approach
Source: STAR Protoc. 2025 Dec 8;6(4):104248. doi: 10.1016/j.xpro.2025.104248 (PMC12741452; doi:10.1016/j.xpro.2025.104248)
Supplement: Data S1. LC-MS MS system work flow [file mmc1.pdf]

Liquid Chromatography System (Ultimate 3000 UHPLC) Setup

Column Configuration

- **Trap Column**: A C18 trap column (heat at 50°C) 300 µm, 5 mm, and 5 µm particles
- The peptides are separated on a 50cm-long EasySpray column (heat at 50°C) (ES803, Thermo Fischer)
- Gradient Program: 2 % to 35 % Buffer B (Buffer A: 5 % DMSO, 0.1 % formic acid; Buffer B: 5 % DMSO, 0.1 % formic acid in acetonitrile) over 60 minutes linear gradient at 250 nL/min flow rate.

Mass Spectrometry Setup (Orbitrap Fusion Lumos Tribrid)

Ion Source

- Analyse eluted peptides on an Orbitrap Fusion Lumos Tribrid (instrument control software v3.3).

MS Parameters

- **MS1 Scan**: Acquire survey scan in the Orbitrap, at resolution 120,000, over range 400–1500 m/z, AGC target of 4e5 and S-lens RF of 30.
- **MS/MS Scan**: Fragment ion spectra (MS/MS) were obtained in the Ion trap (rapid scan mode) with a Quad isolation window of 1.6, 40% AGC target and a maximum injection time of 35 ms, with HCD activation and 28% collision energy. **Dynamic Exclusion**: Enabled 30 s

Calibration

- Regular calibration using standard peptide mix is recommended.

Data Acquisition and Analysis

Software

- Use **Xcalibur** for acquisition for data.

Database Search

- Search against protein databases using UniProt/SwissProt

- Set parameters: enzyme specificity: Trypsin, precursor tolerance (±10 ppm), fragment tolerance (±0.02 Da), fixed modifications (e.g., Carbamidomethylating of cysteine C).
- Searches were performed using the MASCOT

Quantification

- Label-free on experimental design.

Quality Control and Troubleshooting

- **Check Chromatograms**: Look for peak shape, retention time consistency.
- **Baseline Stability**: Minimize noise by using fresh solvents and proper column conditioning.
- **Recalibration**: Perform regularly to maintain mass accuracy.
